# Ketamine for depressive symptoms: A retrospective chart review of a private ketamine clinic

**DOI:** 10.4102/sajpsychiatry.v30i0.2176

**Published:** 2024-02-19

**Authors:** Vidette M. Juby, Saaeda Paruk, Mitsuaki Tomita, Bonga Chiliza

**Affiliations:** 1 Discipline of Psychiatry, College of Clinical Medicine, University of KwaZulu-Natal, Durban, South Africa

**Keywords:** ketamine, intravenous, induction, maintenance, major depression

## Abstract

**Background:**

There is currently no published evidence demonstrating the effectiveness and safety of subanaesthetic doses of ketamine, when administered intravenously as an adjunct treatment for depressive symptoms, in a real world setting in South Africa.

**Aim:**

This retrospective chart review reports the clinical response (change in Patient Health Questionnaire – 7 score) to an initial infusion series of ketamine added to usual treatment, and the pattern of its subsequent maintenance use, for depressive symptoms.

**Setting:**

A private ketamine clinic in Hilton, KwaZulu-Natal.

**Methods:**

The medical records of all patients who attended a private ketamine clinic between August 2019 and 31 May 2021 were retrospectively analysed. Depression symptoms were evaluated using the Patient Health Questionaire-9 (PHQ-9) administered immediately before and 24 h after each treatment. Response was defined as a score decrease of more than 50%.

**Results:**

Among the 154 patients who received ketamine infusions for depression, 67 completed a six infusion initial series, with a response rate of 60.6% and remission rate of 32.4%. Of the 154, 50% no longer experienced any suicidal ideation after treatment and adverse events were uncommon, with 6.2% of infusions requiring intervention for adverse events, mostly nausea. In addition, 48.5% of those who completed the initial series continued to receive maintenance infusions, with no evidence of escalating use or abuse.

**Conclusion:**

Incorporating intravenous ketamine into the existing treatment regimens at a private clinic was associated with reduced acuteness of depression severity and suicidal ideation. This approach appeared safe and tolerable, showing no signs of abuse or dependence.

**Contribution:**

This is the first known naturalistic study reporting on ketamine use for depressive symptoms in South Africa.

## Introduction

Globally, major depressive disorder is among the top 20 causes of disability adjusted life years (DALYs) and the third highest cause of years lost to disability (YLD).^1^ Treatment with anti-depressants can lead to remission in up to 67% of patients, although the majority will require more than one trial of medication, and with each trial, the likelihood of remission decreases.^2^ In the Sequenced Treatment Alternatives to Relieve Depression (STAR*D) trial, the mean time to remission was 6.7 weeks,^3^ which may be difficult for patients to tolerate. The search for an effective anti-depressant that has a rapid onset of action with good tolerability and safety therefore needs to continue.

Ketamine is best known as a dissociative anaesthetic drug and can be administered intravenously, intramuscularly, nasally and orally. However, in 2000, Berman and colleagues published the first study to show that a sub-anaesthetic dose (0.5 mg/kg) of ketamine, administered intravenously over 40 min, resulted in a reduction in depressive symptoms within 24 h when compared with intravenous saline placebo.^4^ Similar results have been replicated since, and a recent meta-analysis of 20 studies suggested that a single dose of ketamine had rapid and significant antidepressant effects, which are sustained by repeated administration of ketamine.^5,6,7^

However, a Cochrane review concluded that although ketamine was more effective than a placebo at reducing symptoms of depression, the effects lasted no longer than a week, the findings being based on poor quality evidence.^8^ Infusions of subanaesthetic doses have also been shown to result in rapid and significant reduction in suicidal ideation scores that may persist for up to a week after infusion.^9,10^ Whether this results in a reduction in suicide attempts and completion is unclear, but proposals have been made for the use of ketamine in the emergency room environment to treat and prevent suicidality.^11^

In addition, some literature supports the use of ketamine as an anxiolytic^12^ as well as to treat post-traumatic stress disorder (PTSD), obsessive compulsive disorder (OCD), substance use disorders, and as an augment in electroconvulsive therapy.^13,14^ Ketamine assisted psychotherapy has also been hailed as a highly effective method for treating anxiety, depression, and heroin addiction.^15,16,17^

Because of concern relating to the development of ketamine abuse, particularly in the club and so-called ‘rave’ scene, there has been caution with regard to its broad use. The dissociative effect or altered ‘psychedelic’ state of mind underpins its abuse and has been suggested to be associated with its antidepressant effects.^18,19,20,21^ A systemic review of side-effects associated with ketamine use in depression reported that acute side-effects are common after treatment, with active assessment and surveillance of these side-effects during trials being inadequate and relying on passive monitoring that is patient reporting. Furthermore, the authors comment that most data about side-effects pertains to short-term or single dosing of ketamine, with little available about cumulative and long-term risks. Most studies report the side effects to be mild and self-limiting, the most common ones being reported were headache, dizziness, dissociation, elevated blood pressure, and blurred vision.^22,23^

While the number of randomised control trials showing that the efficacy and safety of ketamine has increased dramatically since 2000, there is limited evidence demonstrating its real-world effectiveness and safety. The characteristics of participants in trials are often rigorously controlled, for example, single psychiatric diagnoses and no medical comorbidities, whereas real-world treatment of treatment resistant depression would require management to be effective and safe in a more heterogenous population, indicating the need for more naturalistic studies.

A literature search did not reveal any published studies of ketamine infusions for non-anaesthetic uses in sub-Saharan Africa. A survey of 23 medical practitioners in the public sector in the Nelson Mandela Metropole, South Africa, reported that ketamine is primarily used for sedation, analgesia and anaesthesia, and that the anaesthesiologists and medical officers surveyed were not aware of any mental health applications.^24^

This study aimed to describe the clinical response (change in Patient Health Questionaire-9 [PHQ-9] score) to an initial infusion series of ketamine and the pattern of its subsequent maintenance use for depressive symptoms among patients at a private ketamine clinic in KwaZulu-Natal (KZN) province, South Africa. Furthermore, it sought to describe the associations between socio-demographic and clinical variables, and the response to ketamine using the PHQ-9.

## Research methods and design

This study was a naturalistic observational study involving a retrospective chart review at a private ketamine clinic in KZN province (hereafter referred to as ‘the clinic’). The authors were invited by the clinic to analyse the data the clinic had collected since the inception of the clinic. The researchers did not have any input into the procedures followed in the clinic and had no financial or other interest in the clinic.

Patients attend the clinic on an outpatient basis specifically to receive ketamine infusion therapy. Ketamine infusion therapy is the only treatment offered at the clinic. The clinic is actively marketed and advertised publicly. Referral by treating psychiatrists, psychologists and general practitioners is encouraged, but is not a requirement. The ketamine infusions are advertised for the treatment of resistant depression, suicidal ideation, bipolar disorder, OCD, PTSD, postpartum depression, and chronic pain conditions. A general practitioner with emergency medicine training runs the clinic.

Patients are seen at the clinic on an appointment basis, and the clinic provides ketamine infusions to patients of any age. After an initial consultation with the general practitioner, they are required to provide informed consent prior to commencing treatment. The recommended treatment regimen is ketamine administered twice weekly for three weeks totalling six infusions. This is referred to an induction phase or initial series. For the purpose of this study, patients were deemed to have completed the induction phase if they received six infusions with no longer than one week between infusions. The use of six infusions for an induction series is in keeping with previous studies.^25,26,27^ After completion of the initial or induction series, patients may request further infusions as maintenance therapy. The clinic recommends that these maintenance infusions be 2 to 4 weekly, subject to the patient’s request. Maintenance therapy is therefore patient driven, that is, patients call to schedule an infusion when they feel it is needed. No limit is placed on the number of maintenance infusions allowed. For the purpose of this study, all infusions after the first infusion not meeting the criteria of the induction phase and/or initial phase were deemed to be maintenance infusions.

The patients are required to pay cash for each infusion but are given a discounted rate if they pay up front for the induction series.

### Sample

The medical charts of all patients who attended the clinic since its inception in August 2019 until 31 May 2021 were reviewed. Charts were excluded if no ketamine infusions were administered (e.g. the patient did not consent or the doctor did not feel ketamine infusion was indicated or appropriate) or if the indication for infusion was anything other than for depressive symptoms.

### Clinical procedures

Ketamine is administered at an initial dose of 0.5 mg/kg/IV on the first infusion, but may be titrated up according to response and at the clinician’s discretion, the maximal dose being 1 mg/kg/IV. Infusions are given over 40 min, with blood pressure, pulse, oxygen saturation, and respiratory rate being recorded before, during, and 30 min–60 min after the infusion just prior to discharge. Most patients are discharged within 1 h of completing the infusion.

### Outcomes and variables

The clinic uses a proforma data sheet that includes information on socio-demographics, clinical profile and information on each of the induction and maintenance ketamine infusions administered. Information on the patient’s response to treatment is routinely collected on a slightly modified PHQ-9,^28^ which they are requested to complete prior to treatment and 24 h after each infusion. The original PHQ-9 requires rating of symptoms over the prior 2 weeks. The wording of the questionnaire used by the clinic was adapted to refer to the period of time since the last ketamine infusion (which could be as recent at 2 days prior in the case of the induction phase). This measure is widely used in clinical settings and has been validated for use in South African populations.^28,29,30^ The clinic uses the PHQ-9 to monitor response rather than to determine need for treatment.

Response is defined as a greater than 50% reduction in PHQ-9 score, while remission is a post-treatment PHQ-9 score of < 5.^28^ The severity of depression is interpreted from the total PHQ-9 score, which ranges from 0 to 27, a total score of 1–4 corresponded with minimal, 5–9 with mild, 10–14 with moderate, 15–19 with moderately severe, and 20–27 with severe depression. The clinic does not have a minimum score for the administration of ketamine and, with the patient’s consent, the six induction infusions are completed even if remission is achieved prior to the 6th infusion.

Data on heart rate (HR), respiratory rate, blood pressure and oxygen saturation are grouped according to pre- and post-infusion measurements at each infusion for comparison.

### Statistical analysis

The data collected were analysed using STATA version 15.1, StataCorp, College Station, Texas, USA, the results are being summarised using descriptive statistics such as ranges, frequencies, percentages, means, and standard deviation. McNemar’s chi-square test was used to establish associations between socio-demographic variables and the presence of improvement in PHQ-9. Independent samples t-test were used to test for differences in the mean PHQ-9 score between patients who received full course of treatment and those who did not. Dependent t-tests were used to test for differences in PHQ-9 means before and after the ketamine treatment course, the level of significance being set at 0.05.

### Ethical considerations

Ethical approval was obtained from the University of KwaZulu-Natal biomedical research ethics committee and permission to conduct the study from the clinic management. BREC/00002365/2021.

## Results

### Socio-demographic and clinical characteristics

The files of 171 patients who attended the clinic between August 2019 and 31 May 2021 were retrieved. Of these 17 were excluded: 6 did not receive any ketamine infusions, 11 received for an indication other than depressive symptoms namely, pain (*n* = 7), obsessive compulsive disorder (*n* = 3) for generalised anxiety disorder (GAD) (*n* = 1). A total of 154 patient files were used in the analysis of 863 infusions that were administered during the study period.

The mean age of patients was 44 years (standard deviation [s.d.] = 14.8; range 14–77 years), 138 patients (89.6%) had unipolar depression and 16 (10.4%) had bipolar depression (Table 1), while 20 (13%) had previously used ketamine, with one admitted to using it recreationally. Over half of the sample (*n* = 79) had at least one medical comorbidity, the most common being cardiovascular conditions (*n* = 22), diabetes mellitus (*n* = 11), and hypothyroidism (*n* = 10).

**TABLE 1:** Sociodemographic and clinical characteristics (*N* = 154).

Variable	Category	*n*	%
Gender	Male	65	42.2
	Female	89	57.8
Age category (in years)	< 18	5	3.2
	18–24	11	7.1
	25–39	42	27.3
	40–59	68	44.2
	60+	28	18.1
Referral by medical practitioner	Yes	121	78.6
	No	33	21.4
Primary mood disorder diagnosis	Major depressive disorder	138	89.6
	Bipolar	16	10.4
Co-morbid psychiatric conditions	Generalised anxiety disorder	34	22.2
	Post-traumatic stress disorder	14	9.1
	Obsessive compulsive disorder	4	2.6
	Attention deficit hyperactivity disorder	9	5.8
	Substance abuse	9	6.1
Psychiatric medication	Yes	126	82.9
	No	26	17.1
	Antidepressants	120	77.9
	Mood stabilisers	51	33.1
	Antipsychotics	36	23.5
	Benzodiazepines	66	42.9
Prior ketamine use	Yes	20	13.0
	No	133	86.4
	Missing data	1	0.7
Comorbid medical conditions	Yes	79	51.3
	No	75	48.7
	Cardiovascular conditions	22	14.3
	Diabetes mellitus	11	7.1
	Gastro-oesophageal reflux	8	5.2
	Epilepsy	3	1.9
	Hypothyroid	10	6.5
	Asthma and chronic obstructive pulmonary disease	3	1.9
	Urogenital	4	2.6
Depression severity (PHQ-9 score)	Mild (5–9)	3	1.9
	Moderate (10–14)	12	7.8
	Moderate severe (15–19)	39	25.3
	Severe (20–27)	99	64.3
	Missing data	1	0.6

The mean PHQ-9 score prior to treatment was 20.6 (s.d. = 4.66), with the majority of patients falling into the moderately severe (15–19) and severe depression (20–27) categories, according to the PHQ-9 score (25.3% and 64.3%, respectively). Thoughts of suicide and/or self-harm were reported by 85% of the sample, with 40.1% reporting this being present nearly all the time.

### Vital sign monitoring

There was no significant difference between the patients’ respiratory rate, diastolic blood pressure and oxygen saturation before and after each ketamine infusion in the induction series, while the HR and systolic blood pressure (SBP) tended to be numerically higher after each infusion. The difference was statistically significant after the first and fourth infusions for both HR and SBP, and after the fifth infusion for SBP only. After the first infusion, the mean increase in HR was 2.78 beats per minute (bpm) (s.d. = 8.34; *p* < 0.001) and blood pressure 5.17 mmHg (s.d. = 10.91; *p* < 0.001), and after the fourth infusion, the mean increase was 2.25 bpm (s.d. = 9.21 *p* = 0.04) and 9.88 mmHg (s.d. = 13.35; *p* < 0.001), respectively. The mean increase in SBP after the fifth infusion was 11.8 mmHg (s.d. = 12.03; *p* = 0.01).

### Safety and tolerability

Premedication to prevent nausea and vomiting was administered in the form of ondansetron 4 mg intravenously prior to 98 (16.4%) of the 597 infusions during the induction series, and adverse events were reported with 9.5% (*n* = 57) of infusions (*n* = 597). The reported events were nausea and/or vomiting (*n* = 35, 62.4%), ‘unpleasant experiences’ (*n* = 4, 7%), anxiety (*n* = 3, 5%), headache (*n* = 3, 5%), tachycardia (*n* = 1, 1.8%), and itching at infusion site (*n* = 1, 1.8%). Of the 57 adverse events, 37 (6.2% of the 597 infusions) required intervention, one was terminated early because of an ‘unpleasant experience’, the one with a headache received oxygen via face mask, and 35 were given ondansetron 4 mg intravenously during the infusion for nausea and/or vomiting (Table 2).

**TABLE 2:** Tolerability; rates of premedication administered, adverse events reported and interventions required.

Tolerability	Total (*N*)	Yes		No	
		*n*	%	*n*	%
**Infusion 1**	154				
Premedication	-	23	14.90	131	85.1
Adverse event	-	25	16.20	129	83.8
Intervention required	-	18	11.70	136	88.3
**Infusion 2**	117				
Premedication	-	18	15.40	99	84.6
Adverse event	-	8	6.80	109	93.2
Intervention required	-	6	5.13	111	94.9
**Infusion 3**	96				
Premedication	-	16	16.70	80	83.3
Adverse event	-	10	10.40	86	89.6
Intervention required	-	6	6.40	90	93.8
**Infusion 4**	87				
Premedication	-	13	14.90	74	85.1
Adverse event	-	4	4.60	83	95.4
Intervention required	-	2	2.30	85	97.7
**Infusion 5**	74				
Premedication	-	15	20.30	59	79.7
Adverse event	-	3	4.10	71	95.0
Intervention required	-	2	2.70	72	97.3
**Infusion 6**	69				
Premedication	-	13	18.80	56	81.2
Adverse event	-	7	10.10	62	89.9
Treatment required	-	5	7.30	64	92.8

### Clinical response

Of the 154 participants, the PHQ-9 depression response rate (≥50% decrease on the PHQ-9 score) was 50% (*n* = 77) with 25% (*n* = 38) achieving remission (PHQ-9 score < 5). However, when using only the patients who completed all six infusions of the induction phase (*n* = 67), the response rate and remission rate were higher at 70.6% and 32.4%, respectively. Median time to response was 15 days, which coincided with the fourth infusion, with nearly 50% no longer having any suicidal ideation after the induction phase. No baseline socio-demographic or clinical variables were significantly associated with their response; however, a lack of prior ketamine use was associated with remission (*p* = 0.02).

### Pattern of maintenance infusions

Of the 154 participants, 67 (43.5%) completed the full induction series of six ketamine infusions, of whom 33 (48.5%) went on to receive maintenance infusions. Nearly a quarter of participants (*n* = 37, 24%) had only one initial infusion, while 21 (13.6%) who did not complete the full six infusion induction phase went on to receive maintenance infusions. A total of 54 patients received 264 maintenance infusions (mean 4.89). The mean number of days between the last induction infusion and the first maintenance infusion was 65.7 days (s.d. = 79.7), and there was no pattern suggesting decreasing intervals between infusions.

## Discussion

To the best of our knowledge, this is the first naturalistic observational study from South Africa describing the clinical response to an initial infusion series of ketamine and the pattern of subsequent maintenance use for depressive symptoms. The key findings were that 32.4% of participants who completed the induction phase of six infusions reported remission of depressive symptoms and almost 50% reported resolution of suicidal ideation. The response and remission rates of the participants who completed the initial six infusion series were higher than those who had fewer than six infusions (70.6% and 32.4% vs. 50% and 25%, respectively). This difference could suggest that more infusions are needed to achieve response and/or remission. Adverse events occurred in only 9.5% of infusions, with nausea being the most common complaint, while no significant haemodynamic changes relating to pulse and blood pressure were reported.

The response rate for patients who completed the induction phase of six ketamine infusions (*n* = 67) was 70.6%, the response rate being higher than that found in other naturalistic studies, such as that by Sakurai et al.,^31^ Wilkinson et al.,^32^ McInnes et al.,^33^ and Wilkowska et al.^34^ that reported a response rate of 18.3%, 45.5%, 53.6% and 61.5%, respectively. Similar to our study, the patients were unblinded, largely self-referred, and paid for their own treatment out-of-pocket.

Almost 50% of participants in our study reported resolution of suicidal ideation, which is in agreement with the findings of Hietamies et al.^35^ who reported that 49% of subjects with suicidal ideation at baseline no longer reported any suicidal ideation after induction. However, these results are higher than reported in the study by Sakurai and colleagues, who reported that, of the 77% of patients expressing suicidal ideation at the outset, its complete resolution by the end of the induction series occurred only in 17.9%. The differences in outcomes may be because of the participants in their study being more severely treatment resistant than our sample, as evidenced by the high number of antidepressant trials (mean number of failed antidepressant trials 7.4 ± 3.7). In our study, suicidal ideation at baseline appeared to be higher (85%), but treatment resistance could not be assessed as treatment history was not recorded. Of the 154 patients who received ketamine during this study, 21% (*n* = 32) were on no psychiatric medication at the time.

McInnes et al. compiled a retrospective analysis of 537 patients who underwent ketamine induction, defined as 4–8 infusions administered over 7–28 days, and demonstrated a 53.6% response rate and, of the 66.3% with suicidal ideation, 42.7% had a total resolution. The outcome was measured using the PHQ-9 score at 14–31 days post-induction (in our study it was administered the day after infusion), this delay possibly accounting for the difference in response rates as the effect of ketamine may wear off over time.

Diastolic blood pressure, respiratory rate and oxygen saturation were not significantly altered by the ketamine infusion. While both mean SBP and mean HR significantly increased after infusions 1 and 4, and mean SBP after infusion 5, the difference between pre- and post-infusion mean values were moderate: mean increase SBP 5.17 (s.d. = 10.91), 9.88 (s.d. = 13.35) and 11.8 (s.d. = 12.3) mmHg after infusion 1, 4 and 5, respectively. The difference between pre- and post-infusion HR was an increase of 2.78 (s.d. = 8.34) and 2.25 (s.d. = 9.21) BPM after infusions one and four, respectively, and suggests that ketamine infusions do not cause overt cardiovascular instability. It is important to observe that in this naturalistic study, the clinic did not exclude any patients with medical comorbidities and more than 50% of the patients had one or more medical conditions.

Premedication of ondansetron was administered prior to 16.4% of induction infusions, with adverse events being uncommon, occurring at only 57 of the initial infusions (9.5%), and only 6.2% of infusions were accompanied by adverse events that required intervention of any kind. This was in the majority of cases nausea, which required the administration of ondansetron intravenously, and only one infusion was terminated early because of ‘unpleasant effects’. The adverse event rate in our study is much lower than that reported by Sakurai et al., who observed that 44.8% of their participants required ondansetron or prochlorperazine because of nausea during infusions and 35.6% complained of anxiety.^31^ In the study led by Wilkinson, 29.6% of participants experienced mild nausea and were given ondansetron intravenously.^32^ There is no clear explanation for the low rates of adverse event reported in our study; however, it is possible that anxiety and negative experiences were under recorded in the clinical notes.

Less than half of the patients completed the initial six infusion series, with no indication as to whether this was because of intolerability of the treatment, cost or perceived lack of benefit. Dropout rates prior to completion of induction in other similar studies vary widely, from 11.3% to 41.6%.^31,32,36^ While 48.5% of those who completed the initial infusion series went on to receive maintenance infusions, there was no indication of decreasing intervals between the maintenance infusions, which may have suggested dependence and tolerance or abuse.

### Limitations

Our analysis has some major limitations that may have affected the results. Being a retrospective chart review, analysis is dependent on the data collected, with the clinic not collecting data about race, socio-economic status, previous psychiatric history, medication history or previous electroconvulsive therapy. Although this is the first report on ketamine use for depression in South Africa, the missing sociodemographic data prevents comment on the representativeness of the sample. There was also no record of whether the patients were receiving any psychotherapy, which may enhance the effects of the ketamine therapy. The clinic studied does not perform a specific diagnostic interview and many patients are self-referred, meaning that the diagnosis and indication for ketamine infusion may be inaccurate. The change of wording of the PHQ-9 to refer to the time period since the last infusion rather than the prior 2 weeks may have affected the validity and reliability of the tool. The timing of the administration of the PHQ-9, namely just before and 24 h after infusion together may only indicate a short-term response. While the average severity of depression based on the PHQ-9 scores fell in the moderately severe to severe range, more than 20% of participants were not on any psychotropic medication at the time of receiving ketamine infusions, which may suggest that these are not treatment resistant cases, as has been the case in many other studies.

Ketamine infusion commonly results in dissociation, which may introduce bias by way of the expectation of effect. The difficulty with selecting a suitable active control to allow double blinding of studies of ketamine makes controlling for this bias and the placebo effect an ongoing research challenge in this field. Although midazolam has been used as an active control, it is not a dissociative drug and thus patients and clinicians may easily discern which drug they have received.

There were considerable variations in the pattern of ketamine infusion used by participants, with most studies using a six infusion protocol with varying maintenance practices. In our study of 154 patients, only 68 individuals completed the typical six infusion induction phase, 37 only had one infusion, and maintenance infusions varied from a single infusion to 27 for a single individual, which limits the interpretation and/or generalisability of the results. While there are similarities in study design between our study and those of Sakurai and Wilkinson, the absence of demographic data, psychiatric, medical and medication history from the clinic data precludes a more in-depth comparison of study populations and findings.

As the ketamine infusions are funded out-of-pocket, this introduces a source of bias, as only those who can afford the treatment receive infusions as well as a potentially augmented placebo response. Information was lacking on the reason for individual infusion patterns, for example, did they have single infusions as they could not afford a full induction course, or did they not continue treatment because of intolerable side effects or lack of efficacy? Many of these limitations can be addressed in future studies by incorporating more detailed data collection and by eliminating the cost to patient as a potential source of bias.

## Conclusion

Ketamine infusion induction and maintenance as an add on to existing treatment in a private clinic demonstrates antidepressant and antisuicidal effects, with apparent safety and tolerability, and without evidence of overt abuse or dependence. However, longer-term research with more participants would help to elucidate particular populations of patients with depressive symptoms who are likely to respond to this treatment, and whether this response is maintained in the long-term.
